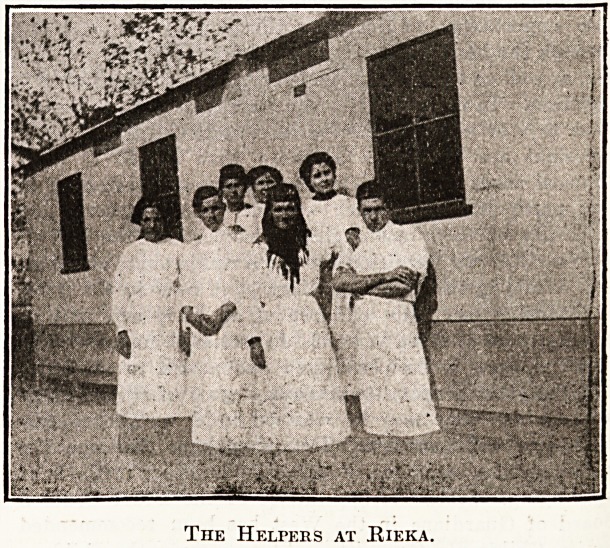# British Workers in Foreign Countries

**Published:** 1913-11-22

**Authors:** 


### BRITISH WORKERS in FOREIGN COUNTRIES.

### II. With the Wounded in Bulgaria.

We were not actually living in hospital, but used to go up there early every day. One morning, about a fortnight after our arrival, on going up to hospital we found the entrance barred by a soldier, who told us that the hospital was in quarantine for a case of cholera, and that no one was allowed in unless intending to remain. Off we dashed to get a few things together, and back we came. Inside, all was confusion among the "Samaritaines," as, of course, they wanted to go home, and none of them had been allowed out. Many were weeping, partly from fear, partly because they had no food. The case was not on our floor, but, of course, every precaution had to be taken. Another sad thing was the lack of accommodation, and one just had to sleep and lie down where one could. The first night I stayed in the wards and the other ladies slept; after that I sampled several kinds of beds—a wooden bench for two nights, which was very hard; then an upright chair; lastly, we discovered some stretchers, which made the most luxurious beds, and we slept like tops. We were allowed, as a great privilege, to sleep in the operating-theatre. I revelled at the shock an English surgeon would have had! But it was a case of "à la guerre comme à la guerre." Fortunately the quarantine lasted only ten days, and I must say we were glad to be free again.

RIEKA, NEAR LAKE SCUTARI, WHERE THERE WERE TEMPORARY HOSPITALS.

### A BULGARIAN CHRISTMAS.

At Christmas everyone seemed determined we should have as good a time as possible, as we were so far from home. We had endless presents, and—what we valued more than anything—a present from our wounded patients of a Bulgarian bread-board and salt-box, like they always use, with their names on the back. The Bulgarian Christmas was thirteen days later than ours, and that I enjoyed even more, as it was so nice getting surprises ready for the men. They taught me to sing their National Anthem, "Schumi Maritza," so that I could take a real part in the rejoicings. The tree was to be on Christmas Eve (January 5), and what a busy day it was getting everything ready! There was a general atmosphere of excitement; the men were in first-rate spirits, busy practising songs for the evening's festivities. We moved those patients who were still in bed into one long room, with the tree at the end, so that they could all get a good view. It was a lovely tree, beautifully decorated. At 6 P.M. the fun began. First, we distributed presents, and then the chief doctor, an Austrian whose knowledge of the language was distinctly limited, got up and made a speech in Bulgarian! This nearly brought the roof down, the men were so delighted. After that one of the patients in bed called out long excited phrases in Bulgarian, with much noise and gesticulation; and suddenly, without any warning, the doctor was hoisted in the air by six of the men and carried triumphantly up and down the room, amidst deafening cheers. It really was a stirring sight! After that the tree was stripped, while the men sang Austrian and Bulgarian songs. They sing on the way to battle, too; we often used to hear them when they were drilling, etc. They are splendidly patriotic; their one idea was to get well quickly, so as to go back and fight. One poor man who had half his hand shot away cheerfully remarked one day, "My hand is a little smaller, but Bulgaria is a little bigger."

### THE MEN IN THE ARMY.

Everyone went to the war—professors, lawyers, carpenters, peasants. It is pitiable to think of all the splendid men they have lost—men who were building up the future of Bulgaria—professors in every branch of science and art. It will be many, many years before they can recover from the war. It was awful to see what mere boys were being drilled and trained ready to be sent to the front; but their heart and soul were in it, and liberty from the Turk meant life in its fullest sense. It was heart-rending to hear what the people had endured and suffered all these years, and the enemy's treatment of the wounded on the battlefield just made one shudder. Several soldiers told me they had kept their arms ready to shoot themselves if they saw a Turk coming. The only Turkish prisoners I had were delightful, and the Bulgarian soldiers were so good to them. Many of the former said they would never forget the kindness they had received.

THE HELPERS AT RIEKA.

The Austrian Mission left Sofia on January 30. We stayed on a week to see if anything further was going to happen, and then decided to go home, as there was not sufficient work to stay for. While on our way back *via* Vienna the war broke out again. The accounts from Montenegro were so terrible and they seemed so badly in need of help we decided to go there. The trip down the Dalmatian coast to Cattaro was lovely; from there we had to drive to Cettinjé, twenty-seven miles, uphill nearly all the way—a wonderful zigzag road up the side of a mountain 3,000 to 4,000 feet high. The view all the way was magnificent, reminding one of a Norwegian fiord. It seemed like spring down in Cattaro (though only the beginning of February), but the higher we went and the nearer we got to Cettinjé the colder it grew.

### Near Lake Scutari.

Reports were true, and we found the place was full of wounded, not only in the public buildings, but also in private houses—even the hotel had housed half a dozen patients. Here also they were badly off for nurses, and so for the next three weeks we had plenty to do. Afterwards for about a fortnight I was working down in a sweet little village near Lake Scutari, called Rieka. It was about six miles from Cettinjé, right down in a valley, and very shut in, so that even in April it seemed almost like summer, with roses out, and figs quite large. Here three movable barracks had been put up and fitted out with beds for fifty patients. The idea was to keep all the worst cases there as they came up the lake from Zogai and other places near the front, while the less severely wounded were sent straight on to Cettinjé. Never shall I forget the arrival of the first lot of wounded one lovely evening about 6 p.m. The whole village turned out to meet them, to see if any were their friends or relations—it was a pathetic sight! And I had hard work to keep the people from crowding into the hospital.

Here I had everything to look after and arrange, besides getting to know my patients well, who were some of the nicest I had had. The fifty beds were never all full, because during that time great pressure was being brought to bear on Montenegro to give up all designs on Scutari, and there was no more fighting. After a fortnight we moved the patients up to Cettinjé, and closed the hospital. About a week later came the news of the surrender of Scutari. Of course, there was great rejoicing. For the time being Cettinjé was mad with joy, and all troubles forgotten in this moment of triumph.

### A River Trip.

The following Sunday I started off with some friends to visit Scutari. It took us nearly two days to get there, as the steamboat service on the lake was all disorganised owing to the war, and there was always a delightful uncertainty as to whether you would get a steamer that day or wait till next—"Neman nischt" (English: "It matters not") as the Montenegrins would say. One soon learns in their country that to-morrow is as good as to-day. The run down the lake was just beautiful. From Reika to the lake the river winds through a fascinating wooded gorge, the water a mass of weeds and lilies, and then when you get into the open, the beautiful snow-topped Albanian mountains burst into view. I was amazed that such a glorious trip could have been left so long unspoilt by tourists. The nearer we got to Scutari the more interesting everything became, and it was hard to realise that we were really seeing the places we had heard of so often; here, an English Red Cross Mission, here a French doctor working, there that desperate fight, etc., and Tarabosh, the magic word that seemed to revive the very dying, the one word in every soldier's mouth. "Let me go back to Tarabosh!" was the eternal cry long before they were well.

### "Back to Tarabosh."

All had the same yearning, the old men even more enthusiastic than the young: "Give me my gun; back to Tarabosh." I am sure all will remember the magnificent attack by the old Montenegrins on the barbed-wire entrenchments half-way up Tarabosh, how they volunteered to dash in and explode the mines. "Old man; he no good; he can die; old man has sons." This was the explanation! What grander sacrifice could have been made! Who could help admiring such magnificent spirit?

No wonder, I thought, as we approached Scutari, that it has been the cause of so long a struggle. Apart from its political and commercial importance, its situation on the borders of the lake is unique. First, one goes through the old and most interesting Turkish quarter, with its endless bazaars and picturesque ramshackle buildings; then on to the more modern part of the town, containing the Government buildings, a Roman Catholic cathedral, and many large Albanian houses enclosed by high walls. Rising above the Turkish quarter is the ancient and imposing citadel—a tower of strength—which looks equal to withstanding many a siege. The town was not nearly so knocked about as I expected. One side of the cathedral was much damaged, and many houses partially destroyed, with large holes knocked in the roof; but, on the whole, the damage was comparatively small. Neither did the people look so thin and ill as I expected after so long a siege. I was told there had always been meat and eggs, but no bread or salt, and as Turks will not eat meat without the former they had suffered considerably. On all sides one heard how splendidly the Montenegrins had behaved since they entered Scutari, and how well organised the food distribution had been, though many were too ill and weak to creep out for food.

### Sympathy for Montenegro.

It was a very hot day when we climbed Tarabosh, a great contrast to the bitter weather during which the soldiers were lying out there day after day on the bare rocks, with little or no shelter. It was horribly realistic to see the barbed-wire entanglements, the guns still in position, and the trenches, some of which were only twenty-five yards distant from those of the enemy. They still seemed full of life—bits of mattresses, sand-bags, papers, old tins, bottles, shells, cartridges, etc., all over the place. It seemed to make even more real the horrors of war, while increasing one's admiration for the combatants.

I was very glad to hear, on my return to England, how much sympathy was felt for Montenegro. Many, like myself, must have longed for her to keep Scutari after her heroic struggles; but it was not to be, and one can only hope that future events will justify the decision of the Powers, and bring to Montenegro the prosperity she so well deserves.

## Figures and Tables

**Figure f1:**
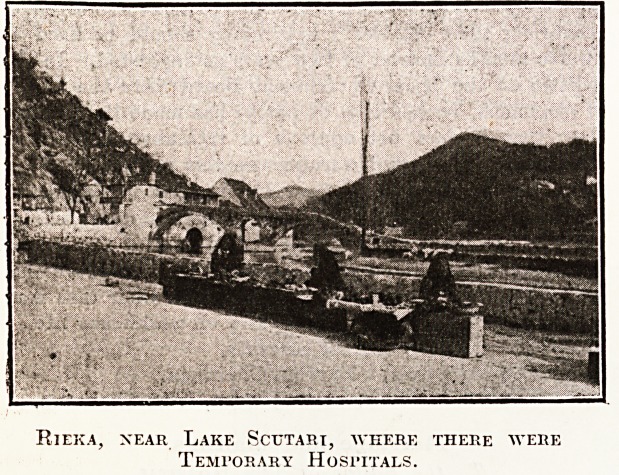


**Figure f2:**